# Association between serum copper, zinc, and selenium concentrations and depressive symptoms in the US adult population, NHANES (2011–2016)

**DOI:** 10.1186/s12888-023-04953-z

**Published:** 2023-07-11

**Authors:** Dong Huang, Shunkai Lai, Shuming Zhong, Yanbin Jia

**Affiliations:** Department of Psychiatry, First Affiliated Hospital of Jinan University, Guangzhou, 510630 China

**Keywords:** Copper, Depression, Micronutrients, NHANES, Selenium, Zinc

## Abstract

**Background:**

Evidence suggests that alterations in serum trace element concentrations are closely associated with mental illness. However, ​studies on the relationship between serum copper, zinc, and selenium concentrations and depressive symptoms are limited and with controversial results. We aimed to investigate the association between serum concentrations of these trace elements and depressive symptoms in US adults.

**Methods:**

Data from the National Health and Nutrition Examination Survey (NHANES) (2011–2016) were used in this cross-sectional study. The Patient Health Questionnaire-9 Items (PHQ-9) was employed to assess depressive symptoms. Multiple logistic regression was performed to determine the relationship between the serum concentrations of copper, zinc, and selenium and depressive symptoms.

**Results:**

A total of 4552 adults were included. Subjects with depressive symptoms had higher serum copper concentrations (123.88 ± 1.87) than those without depressive symptoms (116.99 ± 0.86) (*p* < 0.001). In Model 2, weighted logistic regression analysis showed that the second (Q2) quartile of zinc concentrations (odds ratio [OR] = 1.534, 95% confident interval [CI]: 1.018 to 2.313) were significantly associated with an increased risk of depressive symptoms. Subgroup analysis revealed that the third (Q3) and fourth (Q4) quartiles of copper concentrations (Q3: OR = 2.699, 95% CI: 1.285 to 5.667; Q4: OR = 2.490, 95% CI: 1.026 to 6.046) were also positively associated with depressive symptoms in obese individuals after controlling for all confounders. However, no significant relationship between serum selenium concentrations and depressive symptoms was observed.

**Conclusions:**

Obese US adults with high serum copper concentrations, as well as US adults in general with low serum zinc concentrations, were susceptible to depressive symptoms. Nevertheless, the causal mechanisms underlying these relationships need to be further explored.

**Supplementary Information:**

The online version contains supplementary material available at 10.1186/s12888-023-04953-z.

## Introduction

Depression is a common mental disorder characterized by high disability and mortality, with a lifetime prevalence of 20% [1, 2]. Notably, the number of individuals with depression was estimated to rise by 27.6% globally in 2020 as a result of the coronavirus disease 2019 (COVID-19) pandemic [3]. ​Moreover, depression is a major contributor to the global burden of diseases [4] and is predicted to be the second leading cause of burden of disease by 2030 [5]. The risk of depression in the general population is increasing along with the lack of response of many patients to antidepressant therapy [6]. ​It is, therefore, important to identify the underlying factors associated with depression, which may facilitate the early identification of high-risk population.

A growing body of evidence suggests that an imbalance of trace elements was contributed to the pathogenesis and pathophysiology of multiple mental illness including depression [7, 8]. Copper (Cu), zinc (Zn), and selenium (Se) are essential trace elements that function as cofactors or structural constituents of a large number of enzymes and other important proteins. ​Copper is a key component of ceruloplasmin metalloproteinase and copper/zinc superoxide dismutase, which are crucial for the antioxidant defense system [9]. Copper imbalance may cause oxidative stress and damage neurons, thereby increasing the risk of depression [10]. Additionally, copper influences depression-related neurotransmitters, such as gamma aminobutyric acid (GABA) and glutamate [11–13]. Our previous study also identified a relationship between serum copper concentrations and neurobiochemical metabolism in depressed patients [14]. Regarding zinc, it is a modulator of synaptic activity, neuronal metabolism and plasticity; dysregulated zinc homeostasis has been linked to a number of neurological disorders, including depression [15]. Animal experiments have shown that zinc deficiency could lead to depressive-like behaviors and reduce the antidepressant-like effect, which involved decreased serum zinc concentrations [16, 17]. Low serum zinc concentrations contributed to elevated serum corticosterone levels in rats with depressive-like behaviors, while hyperactivation of the hypothalamic-pituitary-adrenal (HPA) axis seemed to underlie depression [16, 18]. ​Normalizing HPA hyperactivity could improve depressive-like behaviors in chronic unpredictable mild stress (CUMS) rats [19]. Another critical role for zinc is its anti-oxidative and anti-inflammatory function in the central nervous system [20, 21]. Moreover, zinc regulates glutamate homeostasis in a concentration-dependent manner, promoting glutamate release at lower concentrations [22, 23]. It is well known that inflammation, oxidative stress and glutamate homeostasis are mechanistically linked to depression [12, 24]. Furthermore, zinc may also affect depression through its interactions with monoamine neurotransmitters (such as dopamine, serotonin, or norepinephrine) [25]. It is known that selenium and selenium-containing proteins (e.g., glutathione peroxidase) are antioxidants [26, 27]. ​Similarly, selenium has anti-inflammatory effects [28]. An experimental animal study has revealed that selenium-containing protein can ameliorate depressive-like behavior by abrogating inflammation and oxidative stress [29]. ​Furthermore, a recent meta-analysis suggested an inverse association between dietary selenium intake and depression [30]. Accordingly, copper, zinc and selenium have been implicated in the pathogenesis of depression.

It is noteworthy that the concentrations of copper [31], zinc [32], and selenium [33] in peripheral blood are correlated with depression. However, studies on the relationship between peripheral blood concentrations of copper, zinc, and selenium and depression are limited, and the results are controversial. Besides, most studies have concentrated on the relationship between copper and zinc concentrations and depression, but few have investigated selenium concentrations. Prior studies found that patients with depression tended to have higher serum copper concentrations and lower serum zinc concentrations compared to healthy controls [8, 14, 34]. However, other researchers established that serum copper concentrations in depressed patients were equal or lower than in healthy volunteers [35, 36]. Similarly, no significant difference in serum zinc concentrations was also observed between depressed and non-depressed control subjects in other studies [37, 38]. With regard to selenium, while two studies have found no association between serum selenium concentrations and depressive symptomology in Iranian adults and Chinese older adults [39, 40], a study conducted in Australian young adults found a non-linear association [41]. Recently, a meta-analysis found no difference in the serum selenium concentrations between depressed and non-depressed participants [33]. There is, however, a limitation to these studies in terms of sample size. Further, our previous preclinical and clinical studies also suggested that copper induced depressive-like behaviors in rats [42], and that the serum copper concentrations were significantly higher in patients with depression than in normal controls [14, 43]. Nevertheless, external validation by clinical studies with large sample sizes is required to confirm our findings. In addition, a large sample study demonstrated that dietary copper, zinc, and selenium intake were associated with depression [44], and the risk of depression varied by intake levels of these trace elements [30, 45]. However, it is unknown whether the risk of depression also differs by the serum concentrations of these trace elements, as studies have shown that dietary intake of trace elements does not totally reflect their blood concentrations [46, 47]. This means that even though studies have explored the dietary intake of these trace elements, further research into their serum concentrations is warranted.

To address the aforementioned issues, we conducted a population-based cross-sectional study to investigate the association between the serum concentrations of these trace elements (copper, zinc, selenium) and depressive symptoms in the US adults (≥ 20 years) by utilizing data from the National Health and Nutrition Examination Survey (NHANES) 2011–2016.

## Materials and methods

### Study design and population

The NHANES is a cross-sectional population-based survey that utilizes a complex, stratified, multistage sampling design, administered by the National Centers for Health Statics (NCHS). Its purpose is to assess the health and nutritional status of children and adults in the United States. In the NHANES program, household interviews and physical examinations are used to collect data from a nationally representative sample every two years. The survey protocol was approved by the NCHS Ethics Review Board; the informed consent of all survey participants was obtained (https://www.cdc.gov/nchs/nhanes/index.htm).

**Fig. 1:**
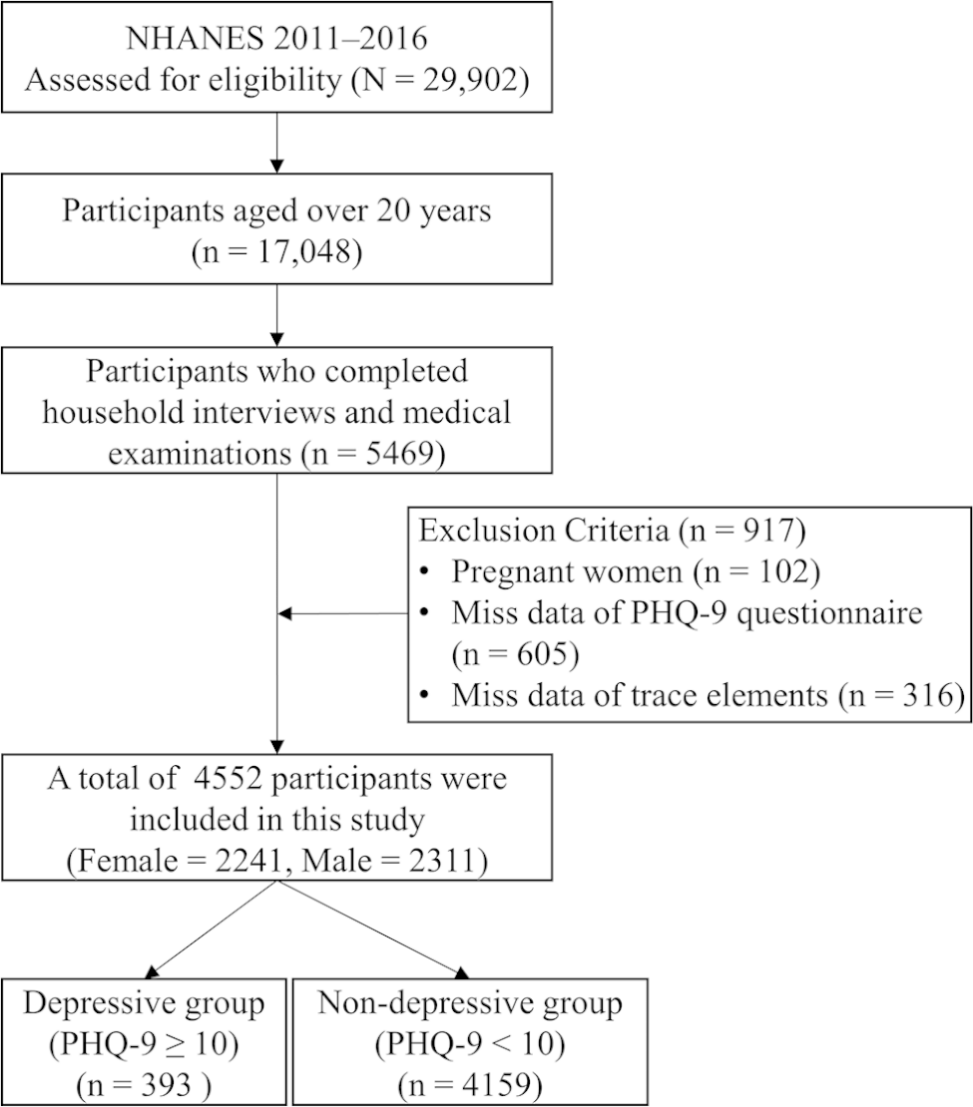
Flow chart for recruiting participants for this study, NHANES (2011–2016). Abbreviations: NHANES, National Health and Nutrition Examination Survey; n, sample size; PHQ-9, the Patient Health Questionnaire-9 Items

Data from three NHANES cycles (2011–2012, 2013–2014, and 2015–2016) which contain information on demographics, depressive symptoms, and serum trace elements were combined and analyzed. A total of 29,902 participants were involved in the NHANES between 2011 and 2016. We then removed 12,854 samples under the age of 20. The remaining 17,048 adult participants aged 20 years and over were selected for analysis. A subsample of 5469 adult participants completed both household interviews and medical examinations was eligible for this study. Female participants who were pregnant (n = 102) were excluded, as pregnancy might have an effect on serum copper and zinc concentrations [48]. We also excluded participants with incomplete depressive symptoms questionnaire (n = 605), and those with missing values of trace elements (n = 316). Finally, 4552 participants aged 20 years and over (2311 male and 2241 female) were included in the current study (Fig. 1).

### Depressive symptoms assessment

The Patient Health Questionnaire-9 Items (PHQ-9) was employed to assess depressive symptoms in NHANES. This instrument is widely used to evaluate individuals’ mental health and screen for depressive symptoms over the past two weeks. The PHQ-9 consists of nine items, each of which is scored from 0 (not at all) to 3 (nearly every day). The overall PHQ-9 score ranges from 0 to 27, with 5, 10, 15, and 20 signifying mild, moderate, moderately severe, and severe depressive symptoms, respectively. The present study defined depressive symptoms as a PHQ-9 score of 10 or above. This cut-off value has an 88% sensitivity and 88% specificity for major depressive symptoms [49].

### Serum trace elements test

Serum copper, zinc and selenium concentrations were measured by inductively coupled plasma dynamic reaction cell mass spectrometry (ICP-DRC-MS). The NHANES laboratory methodology is described in detail at https://wwwn.cdc.gov/Nchs/Nhanes/2015-2016/CUSEZN_I.htm. All results of these serum trace elements match the Division of Laboratory Sciences’ quality control and quality assurance performance criteria for accuracy and precision, similar to the Westgard rules [50]. The lower limit of detection (LLOD) for serum copper, selenium, and zinc are 2.5 µg/dL, 4.5 µg/dL, and 2.9 µg/dL, respectively. For analytes with analytic results below the LLOD, an imputed fill value was placed in the analyte results field. The value is the LLOD divided by the square root of 2 (LLOD/√2).

### Covariates

The demographics characteristics were obtained from household interview and mobile examination center (MEC) interview by trained interviewers using Computer-Assisted Personal Interview (CAPI) system. The present study included age, gender, race (Mexican American, Non-Hispanic White, Non-Hispanic Black, Other Hispanic, and Other race), educational level (less than 9th grade, 9–11 grade, high school graduate/GED or equivalent, some college or AA degree, college or above), marital status (married, widowed, divorced, separated, unmarried and cohabitation), family size (total number of people in the family), annual family income, body mass index (BMI), smoking and alcohol drinking, diabetes and hypertension condition. Alcohol drinking was defined as having 4/5 or more drinks every day. A drink is equivalent to 12 ounces of beer, 5 ounces of wine or 1.5 ounces of liquor. Smoking was described as smoking at least 100 cigarettes in a lifetime. BMI was calculated as weight divided by height squared and categorized as not overweight/obese (BMI < 25 kg/m^2^), overweight (BMI ≥ 25 kg/m^2^ and < 30 kg/m^2^), and obese (BMI ≥ 30 kg/m^2^). The history of hypertension and diabetes stemmed from participants’ self-reported physician diagnoses.

### Statistical analysis

We conducted all statistical analyses using R version 4.1.3 to accommodate the complex sampling design of the NHANES. Three 2-year survey cycles of NHANES (2011–2012, 2013–2014, and 2015–2016) were combined, with new sample weights constructed before analysis (see Supplementary Material). New sample weights were used in all analyses.

Continuous and categorical variables are expressed as mean ± standard deviation (SD) and count (n) with percentage (%), respectively. The missing values of covariates were imputed using multiple imputation. Comparisons of the demographic data between the two groups (depressive symptoms vs. non-depressive symptoms) were performed using either Student’s t-test or chi-square test. Additionally, serum copper, zinc, and selenium concentrations were divided into quartiles (quartile 1 (Q1): < 25%, quartile 2 (Q2): ≥ 25–50%, quartile 3 (Q3): ≥ 50–75%, and quartile 4 (Q4): ≥ 75%). Using the lowest quartile (Q1) as the reference group, univariate and multivariate logistic regression models were utilized to investigate relationships between these trace elements and depressive symptoms. The odds ratio (OR) and 95% confidence interval (CI) were provided. We employed three models in these analyses: Crude Model, Model 1, and Model 2. Crude Model was adjusted for no covariates. Model 1 adjusted for age, gender, and race. Model 2 adjusted for age, gender, race, educational level, marital status, family size, family income, BMI, smoking, alcohol drinking, diabetes, and hypertension. We calculated *P* for trend by entering the median value of each category of serum copper, selenium, and zinc concentrations as continuous variables in the models. As the observed association between serum copper concentrations and depressive symptoms in both Crude Model and Model 1 was no longer significant in Model 2, *P* for interaction between serum copper and the covariates in Model 2 was performed to identify potential moderating variables. Subgroup analyses of these potential moderators would then be conducted to more precisely identify the subgroups that exhibited an association between serum copper concentrations and depressive symptoms in Model 2. Moreover, we performed two sensitivity analyses to verify the stability of the association between serum zinc concentrations and depressive symptoms. First, multilinear regression analysis was performed with PHQ-9 scores as the dependent variable and serum zinc concentrations as the independent variable. Second, an E-value was computed to estimate the effect of unmeasured confounders on the association between serum zinc concentrations and depressive symptoms [51]. The lowest possible E-value is 1. The higher the E-value, the more unmeasured confounding factors are needed to explain away the observed association. All *P* values less than 0.05 (two-sided) were considered statistically significant.

## Results

### Basic characteristics of participants

The detailed characteristics of the study samples are presented in Table 1. Overall, 4552 subjects were included in the presents study, of whom 2311 (50.77%) were male and 2241 (49.23%) female. All subjects were adults above the age of 20: the age of 1986 (43.63%) subjects ranged between 20 and 45 years, and the age of 1841 (40.44%) subjects ranged between 46 and 69 years; the rest of the subjects (15.93%) were over the age of 69. Non-Hispanic whites made up the majority of participants (39.81%), followed by non-Hispanic blacks (21.33%), other races (14.52%), Mexican-Americans (13.55%), and other non-Hispanic (10.79%). The total numbers of drinkers and smokers were 695 (15.27%) and 1994 (43.80%), respectively. In addition, 1678 (36.86%) participants were diagnosed with diabetes and 763 (16.76%) participants with hypertension. Serum copper, zinc, and selenium concentrations were 117.50 ± 28.85 µg/dL, 82.21 ± 15.23 µg/dL, and 130.41 ± 18.65 µg/L, correspondingly.

**Table 1:** Characteristics of participants by depressive status, NHANES (2011–2016). Weighted

	n (%)	Non-depressive group (PHQ-9 < 10, %)	Depressive group (PHQ-9 ≥ 10, %)	*P* value
All participants	4552 (100.00)	4159 (91.37)	393 (8.63)	——
Gender				**0.001**
Male	2311 (50.77)	2152 (93.12)	159 (6.88)	
Female	2241 (49.23)	2007 (89.56)	234 (10.44)	
Age, years				**< 0.001** ^***^
20–45	1986 (43.63)	1853 (93.30)	133 (6.70)	
46–69	1841 (40.44)	1642 (89.19)	217 (11.79)	
> 69	725 (15.93)	682 (94.07)	43 (5.93)	
Age, years (SD)	48.22 (17.03)	48.21 (17.14)	48.32 (15.72)	0.918
Race				**0.028** ^*^
Mexican American	617 (13.55)	572 (92.71)	45 (7.29)	
Other Hispanic	491 (10.79)	433 (88.19)	58 (11.81)	
Non-Hispanic White	1812 (39.81)	1651 (91.11)	161 (8.89)	
Non-Hispanic Black	971 (21.33)	882 (90.83)	89 (9.17)	
Other Race	661 (14.52)	621 (93.95)	40 (6.05)	
Educational level, years				**< 0.001** ^***^
< 9	438 (9.62)	377 (86.07)	61 (13.93)	
9–11	575 (12.68)	491 (85.39)	84 (14.61)	
12	987 (21.68)	896 (90.78)	91 (9.22)	
> 12	2552 (56.06)	2395 (93.85)	157 (6.15)	
Marital status				**< 0.001** ^***^
Married	2275 (49.98)	2141 (94.11)	134 (5.89)	
Widowed	335 (7.36)	298 (88.96)	37 (11.04)	
Divorced	496 (10.90)	421 (84.88)	75 (15.12)	
Separated	134 (2.94)	110 (82.09)	24 (17.91)	
Unmarried	927 (20.36)	842 (90.83)	85 (9.17)	
Cohabitation	385 (8.46)	347 (90.13)	38 (9.87)	
Family size				0.125
1	1071 (23.53)	950 (88.70)	121 (11.30)	
2–4	2536 (55.71)	2340 (92.27)	196 (7.73)	
> 4	945 (20.76)	869 (91.96)	76 (8.04)	
Family income ($)				**< 0.001** ^***^
< 15,000	732 (16.08)	602 (82.24)	130 (17.76)	
15,000–44,999	1823 (40.05)	1639 (89.91)	184 (10.09)	
45,000–99,999	1247 (27.39)	1186 (95.11)	61 (4.89)	
≥ 100,000	750 (16.48)	732 (97.60)	18 (2.40)	
Body mass index (BMI)				**0.029** ^*^
< 25 kg/m^2^	1321 (29.02)	1232 (93.26)	89 (6.74)	
25 to < 30 kg/m^2^	1476 (32.43)	1373 (93.02)	103 (6.98)	
≥ 30 kg/m^2^	1755 (38.55)	1554 (88.55)	201 (11.45)	
Smoking				**< 0.001** ^***^
Yes	1994 (43.80)	1749 (87.71)	245 (12.29)	
No	2558 (56.20)	2410 (94.21)	148 (5.79)	
Alcohol drinking				**< 0.001** ^***^
Yes	695 (15.27)	574 (82.59)	121 (17.41)	
No	3857 (84.73)	3589 (93.05)	268 (6.95)	
Diabetes				**< 0.001** ^***^
Yes	763 (16.76)	654 (85.71)	109 (14.29)	
No	3789 (83.24)	3505 (92.50)	284 (7.50)	
Hypertension				**< 0.001** ^***^
Yes	1678 (36.86)	1472 (87.72)	206 (12.28)	
No	2874 (63.14)	2687 (93.49)	187 (6.51)	
Trace element (SD)				
Copper (Cu, µg/dL)	117.50 (28.85)	116.99 (29.00)	123.88 (26.05)	**< 0.001** ^***^
Selenium (Se, µg/L)	130.41 (18.65)	130.55 (18.64)	128.75 (18.71)	0.122
Zinc (Zn, µg/dL)	82.21 (15.23)	82.32 (15.35)	80.85 (13.60)	0.158

^*^p < 0.05, ^**^p < 0.01, ^***^p < 0.001

Abbreviations: SD: Standard deviation; n, sample size; NHANES, National Health and Nutrition Examination Survey; PHQ-9, the Patient Health Questionnaire-9 Items

The prevalence of depressive symptoms (PHQ-9 ≥ 10) among all participants was 8.63%. Participants who were female (10.44%), smoker (12.29%), alcohol drinker (17.41%), and had a history of diabetes (14.29%) or hypertension (12.28%), were more likely to suffer from depressive symptoms. In addition, depressive symptoms were most prevalent among participants aged 46–69 (11.79%), followed by those aged 20–45 (6.70%) and over 69 (5.93%), with a statistically significant difference (*p* < 0.001). In terms of race, non-Hispanic whites (8.89%) had lower rates of depressive symptoms than non-Hispanic blacks (9.17%) and other Hispanics (11.81%) (*p* = 0.028). Besides, participants with poor education (< 9 years, 13.93%; 9–11 years, 14.61%), low family income (< 15,000 $, 17.76%; 15,000–44,999 $, 10.09%), divorce (15.12%), separation (17.91%), or obesity (11.45%) exhibited statistically significant higher rates of depressive symptoms. The subjects in the depressive group (123.88 ± 26.05 µg/dL) had higher serum copper concentrations than those in the non-depressive group (116.99 ± 29.00 µg/dL) (*p* < 0.001). However, no significant between-group differences (depressive group vs. non-depressive group) were observed in serum zinc and selenium concentrations.

### Regression analysis of the association between serum trace elements and depressive symptoms

The results of the multiple logistic regression analysis of the association between serum copper, zinc, and selenium concentrations and depressive symptoms are displayed in Table 2. Serum copper concentrations in Q3 (OR = 2.070, 95% CI: 1.312 to 3.264) and Q4 (OR = 2.193, 95% CI: 1.320 to 3.645) were significantly related to an increased risk of depressive symptoms in Crude Model (*P* for trend < 0.001). The results remained robust and significant after adjustments for age, gender, and race in Model 1(Q3: OR = 1.925, 95% CI: 1.186 to 3.124; Q4: OR = 1.932, 95% CI: 1.090 to 3.424; *P* for trend = 0.002). However, after adjusting for all covariables in Model 2, these associations vanished (all *p* > 0.05). Surprisingly, we discovered that serum zinc concentrations in Q2 were positively associated with depressive symptoms in all three logistic regression models, with significant and stable results (Crude Model: OR = 1.577, 95% CI: 1.078 to 2.307; Model 1: OR = 1.593, 95% CI: 1.089 to 2.329; Model 2: OR = 1.566, 95% CI: 1.037 to 2.363). In Model 2, the OR for depressive symptoms gradually decreased from the lowest to the highest serum zinc quantiles, but this tendency was not statistically significant (*P* for trend = 0.221). In contrast, no correlations were observed between serum selenium concentrations and depressive symptoms.

**Table 2:** Weighted odds ratios (95% confidence intervals) for depressive symptoms across quartiles of copper, zinc, and selenium, NHANES (2011–2016)

	Crude Model	Model 1	Model 2
Cu (µg/dL)			
Q1 (< 99.20)	ref	ref	ref
Q2 (99.20–114.55)	1.137 (0.631, 2.046)	1.099 (0.595, 2.033)	0.876 (0.449, 1.711)
Q3 (114.56–133.60)	**2.07 (1.312, 3.264)** ^**^	**1.925 (1.186, 3.124)** ^**^	1.149 (0.676, 1.955)
Q4 (>133.60)	**2.193 (1.32, 3.645)** ^**^	**1.932 (1.090, 3.424)** ^*^	1.037 (0.539, 1.993)
*P* for trend	< 0.001	0.002	0.716
Se (µg/L)			
Q1 (< 118.40)	ref	ref	ref
Q2 (118.40–128.40)	0.866 (0.608, 1.232)	0.894 (0.625, 1.281)	1.003 (0.679, 1.483)
Q3 (128.41–139.80)	0.727 (0.459, 1.151)	0.768 (0.481, 1.226)	0.9 (0.544, 1.489)
Q4 (>139.80)	0.91 (0.655, 1.265)	1 (0.705, 1.418)	1.201 (0.817, 1.765)
*P* for trend	0.548	0.924	0.430
Zn (µg/dL)			
Q1 (< 71.30)	ref	ref	ref
Q2 (71.30–80.40)	**1.577 (1.078, 2.307)** ^*^	**1.593 (1.089, 2.329)** ^*^	**1.566 (1.037, 2.363)** ^*^
Q3 (80.41–90.40)	0.961 (0.562, 1.644)	0.992 (0.583, 1.686)	1.012 (0.564, 1.816)
Q4 (> 90.40)	0.870 (0.581, 1.301)	0.942 (0.623, 1.424)	0.871 (0.555, 1.369)
*P* for trend	0.129	0.285	0.221

Crude Model is the unadjusted model

Model 1 adjusted for age, gender and race

Model 2 adjusted for age, gender, race, educational level, marital status, family size, family income, BMI, smoking, alcohol drinking, diabetes and hypertension

^*^p < 0.05, ^**^p < 0.01, ^***^p < 0.001

Abbreviations: Q1, the first quartile; Q2, the second quartile; Q3, the third quartile; Q4, the fourth quartile; Cu, copper; Zn, zinc; Se, selenium; NHANES, National Health and Nutrition Examination Survey; ref, reference

### Subgroup analysis

To analyze for the potential factors influencing the correlation between serum copper concentrations and depressive symptoms, we conducted subgroup analysis. First of all, the interactions between serum copper concentrations and all covariates included in Model 2 were calculated, and the results are shown in Table S1. In Model 2, we found that the interactions between copper and BMI, smoking, or hypertension were related to depressive symptoms. Therefore, we further conducted subgroup analysis of the association between depressive symptoms and serum copper concentrations stratified by the BMI, smoking, and hypertension. In the subgroup analysis of BMI, individuals with serum copper concentrations in Q3 and Q4 had the highest OR of depressive symptoms when their BMI was higher than 30 kg/m^2^ in Model 2 (Q3: OR = 2.699, 95% CI: 1.285 to 5.667; Q4: OR = 2.490, 95% CI: 1.026 to 6.046; *P* for trend = 0.028) (Table 3). However, no significant results were observed in Model 2 in the subgroup analysis of the smoking and hypertension (all *p* > 0.05) (Table S2, S3). These findings suggested that BMI might mainly affect the copper-depressive symptoms relationship.

**Table 3:** Weighted odds ratios (95% confidence intervals) for depressive symptoms by subgroup of BMI according to quartiles of serum copper concentrations, NHANES (2011–2016)

	Crud Model	Model 1	Model 2
< 25 kg/m^2^			
Cu (µg/dL)			
Q1 (< 99.20)	ref	ref	ref
Q2 (99.20–114.55)	2.028 (0.878, 4.687)	**2.609 (1.076, 6.327)** ^*^	1.825 (0.703, 4.732)
Q3 (114.56–133.60)	1.230 (0.483, 3.133)	1.730 (0.624, 4.797)	0.735 (0.228, 2.365)
Q4 (> 133.60)	1.377 (0.611, 3.104)	2.020 (0.769, 5.309)	0.877 (0.325, 2.363)
*P* for trend	0.683	0.221	0.348
25 to < 30 kg/m^2^			
Cu (µg/dL)			
Q1 (< 99.20)	ref	ref	ref
Q2 (99.20–114.55)	0.656 (0.293, 1.470)	0.616 (0.269, 1.409)	0.446 (0.185, 1.075)
Q3 (114.56–133.60)	1.572 (0.720, 3.432)	1.422 (0.622, 3.252)	0.799 (0.355, 1.798)
Q4 (> 133.60)	1.139 (0.525, 2.475)	0.928 (0.365, 2.361)	0.528 (0.212, 1.311)
*P* for trend	0.336	0.728	0.313
≥ 30 kg/m^2^			
Cu (µg/dL)			
Q1 (< 99.20)	ref	ref	ref
Q2 (99.20–114.55)	1.216 (0.601, 2.460)	1.089 (0.531, 2.236)	0.979 (0.437, 2.196)
Q3 (114.56–133.60)	**4.032 (2.186, 7.438)** ^***^	**2.997 (1.535, 5.849)** ^**^	**2.699 (1.285, 5.667)** ^*^
Q4 (> 133.60)	**4.779 (2.558, 8.930)** ^***^	**3.113 (1.410, 6.869)** ^**^	**2.490 (1.026, 6.046)** ^*^
*P* for trend	< 0.001	0.004	0.028

Crude Model is the unadjusted model

Model 1 adjusted for age, gender and race

Model 2 adjusted for age, gender, race, educational level, marital status, family size, family income, smoking, alcohol drinking, diabetes and hypertension

^*^p < 0.05, ^**^p < 0.01, ^***^p < 0.001

Abbreviations: BMI, body mass index; Q1, the first quartile; Q2, the second quartile; Q3, the third quartile; Q4, the fourth quartile; Cu, copper; NHANES, National Health and Nutrition Examination Survey; ref, reference

### Sensitivity analysis

To validate the robustness of the relationship between serum zinc concentrations and depressive symptoms, two sensitivity analyses were performed. The main reasons for these analyses were as follows. On the one hand, dichotomizing PHQ-9 scores into depressive symptoms and non-depressive symptoms just demonstrated a sensitivity of approximately 88%. However, utilizing a linear regression model with PHQ-9 scores as a continuous variable may enhance the reliability of the outcomes. On the other hand, despite controlling for numerous covariates in Model 2, the potential influence of unmeasured covariates on the outcomes remained unclear. To estimate the effect of unmeasured or unknown confounders, the E-value was calculated. Controlling for all covariates, there was an inverse correlation between PHQ-9 scores and serum zinc concentrations (ß = -0.013, 95% CI: -0.022 to -0.005, *p* = 0.004) (Table S4), demonstrating that depressive symptoms might be more likely to be detected in subjects with low serum zinc concentrations. In addition, the E-value for the effect estimate (confidence interval) of the relationship between low serum concentrations of zinc (Q2) and depressive symptoms in Model 2 was 1.81 (1.15). The E-value suggested that our results were robust unless an unmeasured confounder had a relative risk greater than 1.81 with both serum zinc concentrations and depressive symptoms.

## Discussion

To our best knowledge, few studies have investigated the associations between serum copper, zinc, and selenium concentrations and depressive symptoms. This is the first study to investigate the association of these serum trace elements with depressive symptoms in US adult population using NHANES data (2011–2012, 2013–2014, and 2015–2016). Our results demonstrated two main findings. First, serum copper concentrations were elevated in US adults with depressive symptoms, and only in obese individuals were the two highest quartiles (Q3 and Q4) of copper concentrations associated with an increased risk of depressive symptoms. Second, lower serum zinc concentrations (Q2) were also consistently and positively associated with depressive symptoms, although no differences were found in serum zinc concentrations between the depressive and non-depressive groups. These findings provide evidence for the relationships of serum copper and zinc concentrations with depressive symptoms.

Our study revealed that the serum copper concentrations in the depressive group were higher than those in the non-depressive group, which was consistent with our earlier finding [14]. Similarly, a recent meta-analysis based on observational research also established higher serum copper concentrations in patients with depression [31]. Besides, copper exposure could induce depressive-like behavior in rat model [42, 52]. A complex mechanism of interaction might exist between serum copper and depressive symptoms. On the one hand, depression has been identified as a pro-inflammatory state [53]. It activates the inflammatory response system [54] and may further promote elevated levels of serum copper [55, 56]. On the other hand, depression is strongly associated with oxidative stress. Evidence implies that patients with depression have excessive levels of reactive oxygen species (ROS), accompanied by elevated superoxide dismutase (SOD) activity [10]. ​Besides, copper is not only a component of copper-zinc superoxide dismutase (Cu/Zn-SOD), but also can regulate Cu/Zn-SOD activity [57]. Meanwhile, copper and its complexes are also known to have antioxidant activities [58]. Thus, elevated serum copper concentrations may be related to increased antioxidant activity in depressed patients. In addition, our previous investigation showed that the mRNA expression levels of ATPase copper-transporting alpha (ATP7A) decreased in patients with major depression [59]. ​ATP7A is known to regulate copper homeostasis, and its abnormal expression may increase serum copper concentrations. In contrast, excessive copper exposure could also alter the levels of many cytokines and cause inflammatory responses [60]. Notably, peripheral inflammation was found to increase the permeability of the blood-brain barrier (BBB) [61], resulting in disrupted brain homeostasis and depression. Otherwise, excessive peripheral blood copper could directly destroy BBB [62], increasing brain copper levels as copper enters the brain mainly through BBB [63]. In turn, excessive brain copper catalyzed the formation of ROS [9, 64], increased the neurotoxic effects of oxidative stress, and induced neuronal oxidative damage [65], which could contribute to depression [10]. Additionally, copper release was associated with N-methyl-D-aspartate (NMDA) receptor activation [66]. Our previous research showed that memantine (NMDA receptor antagonist) treatment not only decreased serum copper levels, but also improved the depressive-like behaviors induced by corticosterone and copper [42]. That is, glutamine activity may partially explain the relationship between elevated serum copper concentrations and depressive symptoms. Copper also binds to serotonin and induces oxidation and structural modification in serotonin, especially at its high concentrations, ultimately resulting in neurotoxicity and serotonergic dysfunction [67–69]. Hence, serotonergic system dysregulation may be related to depressive symptoms induced by high copper concentrations [70]. High serum copper concentrations may also induce depression by influencing neurobiochemical metabolism [14].

Moreover, this study revealed that obese individuals with high copper concentrations were more likely to have depressive symptoms. Previous studies have found that obesity was associated with higher depression prevalence [71, 72] and obesity prevalence was associated with depression severity [73, 74]. In addition, BMI was positively linked not only to depression [75] but also to serum copper concentrations [76], especially in regards to the association of the increased odds of obesity with elevated serum copper concentrations [77]. Taken together, obesity may be a moderator in the relationship between depression and high copper concentrations. Notably, depression was accompanied by inflammation [54]; inflammatory processes could also lead to copper accumulation [78]. Whereas, obese individuals tended to be in an inflammatory state [79]. Obesity, thus, may induce depression by affecting copper metabolism through inflammation [71, 80]. Meanwhile, the interaction of inflammatory and oxidative stress may play an important role in this process. Copper, increased by obesity, could directly raise the levels of reactive oxygen species (ROS) and reduced the activities of antioxidant enzymes, further activating the microglial ROS/nuclear factor-kappa B (NF-κB) pathway to secrete inflammatory products, leading to neuroinflammatory response and neuronal apoptosis [81, 82], thereby inducing depression [83]. ​Furthermore, cortisol reactivity mediated the depression-obesity relationship [84]. The interplay between cortisol and inflammation might also be the underlying mechanism for the relationship between copper and depression in obese subjects [85]. Thus, it is possible that maintaining relatively low serum copper concentrations in obese populations may be beneficial for reducing the risk of depressive symptoms, but further investigations are needed to provide evidence to this speculation. However, weight control may be more conducive to physical and mental health. Nevertheless, the underlying causal mechanism of depressive symptoms in association with serum copper concentrations in obese subjects is still unclear.

In addition, the current study suggested that depressed subjects appeared to have lower serum zinc concentrations than non-depressed subjects, but this difference was not statistically significant. This observed trend was similar to previous findings of a significant decrease in serum zinc levels in patients with depression [35, 86, 87]. There are several possible reasons for this unremarkable discrepancy of the current study. First, there was a difference in the study subjects. In the present study, participants with depressive symptoms were not depressed patients; their depressive symptoms might be milder in severity and shorter in duration than patients with depression. A negative correlation was also found between serum zinc concentrations and depressive symptoms severity in the present study. Therefore, we speculated that serum zinc concentrations did not fall significantly because the present study subjects were not patients diagnosed with major depression with major depression with severe depressive symptoms. Second, there might be regional and ethnic differences in subjects between studies. Only US populations were included in the present study. Genetic differences might exist among races, and differences in dietary structure and risk of zinc exposure might exist among regions. Third, the sample size in the present study varied widely between depressive group and non-depressive group.

Furthermore, we noticed that lower serum zinc concentrations were associated with depressive symptoms. This result was robust in all three regression models. Our findings were consistent with the ones of previous studies [86, 87], which indicated that individuals ​with lower serum zinc concentrations within the physiologic range may be more susceptible to depressive symptoms. Collectively, lower serum zinc concentrations may be a risk factor of depressive symptoms. Zinc was considered to interact with the serotonin system [88] and BDNF [89]. Hence, lower serum zinc concentrations could compromise serotonin and BDNF activity and diminish neurogenesis, which may be the pathophysiology of depression [25]. Zinc is a modulator of excitatory (glutamate) and inhibitory (GABA) neurotransmitters [90]: zinc binds to GluN2A subunit via zinc transporter 1 and inhibits N-methyl-d-aspartic acid (NMDA) receptor function [91]; zinc activates the zinc-sensing receptor GPR39 to regulate glutamate and GABA, maintaining the brain’s excitatory-inhibitory balance [23]. Consequently, ​reduced zinc concentrations may trigger glutamate release and elicit neuronal excitotoxicity, which contributes to depression [92]. Notably, the synergistic interaction among low serum zinc concentrations, GPR39, BDNF, and serotonergic system may be an underlying mechanism of depression [88, 89]. Additionally, it has been demonstrated that inflammation and oxidative stress are implicated in the pathophysiology of depression [24, 93]. Zinc deficiency could activate the immune-inflammatory response system. Low serum zinc concentrations were usually accompanied by raised immuno-inflammatory indicators like CD4+/CD8 + T-cell ratio and interleukin 6 in depressed patients [94, 95]. Zinc deficiency also triggered oxidative stress, further activating oxidant-sensitive transcription factors such as NF-κB and activator protein-1 (AP-1), thereby causing DNA damage and neuronal apoptosis, ultimately leading to depression [96–98]. Therefore, low serum zinc concentrations may cause depression through the interaction of inflammatory cytokines and oxidative products. ​Moreover, HPA axis hyperactivity has been demonstrated in depression and is associated with decreased serum zinc concentrations [16, 99]. There is also a close association between cortisol concentrations and immune/inflammatory markers in patients with depression [100, 101]. ​Thus, low zinc concentrations may promote depression via the interplay between immune/inflammatory and HPA axis functions. Besides, an animal study found that zinc deficiency caused phospholipid-protein imbalance leading to depression due to the effect on phospholipids and proteins [102]. Thus, maintaining relatively high serum zinc concentrations within the normal range may be associated with a decreased risk of depressive symptoms. Dietary zinc intake or zinc supplementation may also help improve depressive symptoms risk [103, 104]. However, the causal mechanisms connecting low serum zinc concentrations and depressive symptoms remain elusive.

The present study did not find group differences in serum selenium concentrations between individuals with and without depressive symptoms, nor an association between serum selenium concentrations and depressive symptoms. These findings were in agreement with previously reported results [33, 105]. Accordingly, there is insufficient evidence to support an association between selenium status and depressive symptoms, and further studies are needed. Nevertheless, selenium, serving as an antioxidant, can help protect the central nervous system from free radical damage [106]; selenium supplementation could alleviate depressive symptoms [107].

Several limitations exist in this study. First, this study was cross-sectional, and, therefore, causal conclusions could not be drawn. Although we provided explanations of the biological mechanisms in the discussion section, animal trials and prospective cohort studies should be conducted to confirm the causal direction of the relationship between serum copper or zinc levels and depressive symptoms. Second, it is still possible that residual confounding factors (e.g., dietary or physical activity) have influenced our results, although a large number of covariates have been controlled. Third, no multiple comparisons correction was applied to the copper interaction test as the analyses were exploratory in nature. Finally, the PHQ-9 is a depression measurement scale rather than a diagnostic instrument, which can be employed for the assessments of depressive symptoms rather than the diagnosis of depression. We also could not exclude the presence of individuals diagnosed with depression, nor did we know the course of depressive symptoms over time. Future studies could confirm our findings by recruiting large samples of participants with depression as well as using a depression assessment scale with better reliability and validity, such as the Hamilton Depression Rating Scale.

## Conclusions

In conclusion, our results suggested that serum copper concentrations were elevated among US adults with depressive symptoms. ​Meanwhile, serum copper concentrations above the general adult population mean were associated with an increased risk of depressive symptoms in obese adults. Additionally, US adults in general with low serum zinc concentrations were more prone to have depressive symptoms. However, direct evidence on the relationship between serum selenium concentrations and depressive symptoms was lacking. Causal associations between the serum copper or zinc concentrations and depressive symptoms and their detailed mechanisms require further prospective human and animal studies.

## Electronic supplementary material

Below is the link to the electronic supplementary material.

Supplementary Material 1

## Data Availability

The datasets generated and analyzed during the current study are available in the NHANES repository, https://www.cdc.gov/nchs/nhanes/.
