# Supplementary material for: Association between serum copper, zinc, and selenium concentrations and depressive symptoms in the US adult population, NHANES (2011–2016)
Source: BMC Psychiatry. 2023 Jul 11;23:498. doi: 10.1186/s12888-023-04953-z (PMC10337172; doi:10.1186/s12888-023-04953-z)
Supplement: Supplementary file 1 — Supplementary Material 1 [file 12888_2023_4953_MOESM1_ESM.docx]

**Supplementary material**

**Association between serum copper, zinc, and selenium concentrations and depressive symptoms in the US adult population, NHANES (2011–2016)**

**Contents**

**Content 1:** Calculation of the new sample weights.

**Content 2:** **Table S1.** The interaction between covariates and serum copper concentrations.

**Content 3:** **Table S2.** Weighted odds ratios (95% confidence intervals) for depressive symptoms by subgroup of smoking according to quartiles of serum copper concentrations, NHANES (2011–2016).

**Content 4:** **Table S3.** Weighted odds ratios (95% confidence intervals) for depressive symptoms by subgroup of hypertension according to quartiles of serum copper concentrations, NHANES (2011–2016).

**Content 5:** **Table S4.** Association between serum zinc concentrations and PHQ-9 score, NHANES (2011–2016). Weighted.

**Content 1. Calculation of the new sample weights.**

According to NHANES requirement, new multi-year sample weights should be computed when combining two or more two-year cycles. The procedure was as following: first, we selected the weights of the smallest sub-population that included all variables of interest, which were the 2-year subsample weights of serum trace element (WTSA2YR). Second, we constructed a new 6-year weight by taking one-third of WTSA2YR after combining the three two-year cycles (2011–2012, 2013–2014, and 2015–2016). More details on the selection and construction of sample weights can be found on the NHANES website: <https://wwwn.cdc.gov/nchs/nhanes/tutorials/weighting.aspx>

Table S1. The interaction between covariates and serum copper levels.

| *P* for interaction ^a^ | copper |
| --- | --- |
| Gender  Age  Race  Educational level  Marital status  Family size  Family income  Body mass index (BMI)  Smoking  Alcohol drinking  Diabetes  Hypertension | 0.459  0.620  0.152  0.148  0.844  0.876  0.157  **0.007^**^**  **0.006^**^**  0.154  0.441  **0.037^*^** |

^a^, All interaction analyses were conducted in Model 2.

^*^*p* < 0.05, ^**^*p* < 0.01.

Table S2. Weighted odds ratios (95% confidence intervals) for depressive symptoms by subgroup of smoking according to quartiles of serum copper concentrations, NHANES (2011–2016).

|  | Crud Model | Model 1 | Model 2 |
| --- | --- | --- | --- |
| Smoking  Cu (µg/dL)  Q1 (< 99.20)  Q2 (99.20–114.55)  Q3 (114.56–133.60)  Q4 (> 133.60)  *P* for trend  Non-smoking  Cu (µg/dL)  Q1 (< 99.20)  Q2 (99.20–114.55)  Q3 (114.56–133.60)  Q4 (> 133.60)  *P* for trend | ref  1.164 (0.568, 2.388)  **2.331 (1.307, 4.160) ^**^**  **2.572 (1.381,** **4.792) ^**^**  < 0.001  ref  0.909 (0.427, 1.937)  1.257 (0.666, 2.372)  1.651 (0.945, 2.883)  0.038 | ref  1.073 (0.510, 2.259)  **1.952 (1.076, 3.541) ^*^**  1.884 (0.963, 3.683)  0.005  ref  0.884 (0.396, 1.972)  1.180 (0.587, 2.375)  1.562 (0.764, 3.192)  0.126 | ref  0.990 (0.450, 2.174)  1.468 (0.772, 2.792)  1.227 (0.588, 2.559)  0.375  ref  0.750 (0.321, 1.749)  0.777 (0.357, 1.692)  0.836 (0.371, 1.883)  0.786 |

Model 1 adjusted for age, gender and race.

Model 2 adjusted for age, gender, race, educational level, marital status, family size, family income, body mass index, alcohol drinking, diabetes and hypertension.

^*^*p* < 0.05, ^**^*p* < 0.01.

Abbreviations: Q1, the first quartile; Q2, the second quartile; Q3, the third quartile; Q4, the fourth quartile; Cu, copper; NHANES, National Health and Nutrition Examination Survey; ref, reference.

Table S3. Weighted odds ratios (95% confidence intervals) for depressive symptoms by subgroup of hypertension according to quartiles of serum copper concentrations, NHANES (2011–2016).

|  | Crud Model | Model 1 | Model 2 |
| --- | --- | --- | --- |
| Hypertension  Cu (µg/dL)  Q1 (< 99.20)  Q2 (99.20–114.55)  Q3 (114.56–133.60)  Q4 (> 133.60)  *P* for trend  Non-hypertension  Cu (µg/dL)  Q1 (< 99.20)  Q2 (99.20–114.55)  Q3 (114.56–133.60)  Q4 (> 133.60)  *P* for trend | ref  1.183 (0.519, 2.696)  2.216 (1.000, 4.910)  **2.484 (1.154,** **5.344) ^*^**  0.002  ref  1.058 (0.501, 2.235)  1.782 (0.904, 3.514)  1.829 (0.937, 3.568)  0.025 | ref  1.195 (0.509, 2.810)  1.964 (0.880, 4.385)  2.042 (0.900, 4.633)  0.027  ref  1.031 (0.474, 2.243)  1.675 (0.819, 3.424)  1.628 (0.742, 3.572)  0.118 | ref  0.923 (0.370, 2.304)  1.205 (0.537, 2.703)  1.116 (0.494, 2.521)  0.609  ref  0.872 (0.384, 1.979)  1.018 (0.485, 2.139)  0.963 (0.424, 2.190)  0.978 |

Model 1 adjusted for age, gender and race.

Model 2 adjusted for age, gender, race, educational level, marital status, family size, family income, body mass index, smoking, alcohol drinking, and diabetes.

^*^*p* < 0.05.

Abbreviations: Q1, the first quartile; Q2, the second quartile; Q3, the third quartile; Q4, the fourth quartile; Cu, copper; NHANES, National Health and Nutrition Examination Survey; ref, reference.

Table S4. Association between serum zinc concentrations and PHQ-9 score, NHANES (2011–2016). Weighted.

|  | ß (95% CI) | t | *P* value |
| --- | --- | --- | --- |
| Crude Model | -0.015 (-0.024, -0.006) | -3.287 | 0.002 |
| Model 1 | -0.011 (-0.020, -0.003) | -2.610 | 0.012 |
| Model 2 | -0.013 (-0.022, -0.005) | -3.107 | 0.004 |

Crude Model was the unadjusted model.

Model 1 adjusted for age, gender and race.

Model 2 adjusted for age, gender, race, educational level, marital status, family size, family income, BMI, smoking, alcohol drinking, diabetes and hypertension.
